# Brain Functional Plasticity Driven by Career Experience: A Resting-State fMRI Study of the Seafarer

**DOI:** 10.3389/fpsyg.2017.01786

**Published:** 2017-10-11

**Authors:** Nizhuan Wang, Weiming Zeng, Yuhu Shi, Hongjie Yan

**Affiliations:** ^1^Laboratory of Digital Image and Intelligent Computation, College of Information Engineering, Shanghai Maritime University, Shanghai, China; ^2^Neuroimaging Laboratory, School of Biomedical Engineering, Health Science Center, Shenzhen University, Shenzhen, China; ^3^Department of Neurology, Affiliated Lianyungang Hospital of Xuzhou Medical University, Lianyungang, China

**Keywords:** resting-state fMRI, brain plasticity, career experience, seafarer, dynamic functional connectome

## Abstract

The functional connectome derived from BOLD resting-state functional magnetic resonance imaging data represents meaningful functional organizations and a shift between distinct cognitive states. However, the body of knowledge on how the long-term career experience affects the brain’s functional plasticity is still very limited. In this study, we used a dynamic functional connectome characterization (DBFCC) model with the automatic target generation process K-Means clustering to explore the functional reorganization property of resting brain states, driven by long-term career experience. Taking sailors as an example, DBFCC generated seventeen reproducibly common atomic connectome patterns (ACP) and one reproducibly distinct ACP, i.e., ACP14. The common ACPs indicating the same functional topology of the resting brain state transitions were shared by two control groups, while the distinct ACP, which mainly represented functional plasticity and only existed in the sailors, showed close relationships with the long-term career experience of sailors. More specifically, the distinct ACP14 of the sailors was made up of four specific sub-networks, such as the auditory network, visual network, executive control network, and vestibular function-related network, which were most likely linked to sailing experience, i.e., continuously suffering auditory noise, maintaining balance, locating one’s position in three-dimensional space at sea, obeying orders, etc. Our results demonstrated DBFCC’s effectiveness in revealing the specifically functional alterations modulated by sailing experience and particularly provided the evidence that functional plasticity was beneficial in reorganizing brain’s functional topology, which could be driven by career experience.

## Introduction

### Functional Plasticity and Career Experience

One important issue in cognitive neuroscience concerns the relationship between brain’s functional plasticity and individually extensive career training or long-term work experience. In recent years, many studies have demonstrated that blood-oxygen-level-dependent (BOLD) functional magnetic resonance imaging (fMRI) is a powerful modality to help reveal the neural correlates of cognitive processes in different conditions, such as undergoing a cognitive task, resting-states (Calhoun et al., 2008; Wang et al., 2012, 2013, 2016b; Liu et al., 2013; Ren et al., 2014; Jing et al., 2015; Shi et al., 2015a; Tang et al., 2015; Wang N. et al., 2015) or mental disorders, including psychological subhealth (Shi et al., 2015b), autism spectrum disorder (Ambrosino et al., 2014), dementia (Rytty et al., 2013), and schizophrenia (Du et al., 2015). Recently, it has also been shown that the resting-state functional connectivity in specific regions is modulated by individual behaviors (Hampson et al., 2006), extensive learning (Albert et al., 2009; Tung et al., 2013), and experiences (Jeong et al., 2006; Orr et al., 2014; Wang L. et al., 2015; Shen et al., 2016). Specifically, Shen et al. (2016) noted that there is a significant link between the changes in the time-dependent aspects of resting-state functional connectivity within the vigilance network and long-term driving experiences. Furthermore, Hervais-Adelman et al. (2015) revealed that brain’s functional plasticity is associated with the emergence of expertise in extreme language control by exploring the functional response of participants with simultaneous interpretation training. Recently, Yang et al. (2016) demonstrated that the functional connectivity strength among certain paired resting-state networks has the significant changes regarding two resting-state conditions of the professional composers’ brain states, i.e., the one before composition task, and the other after composition task. Based on the aforementioned studies, the brain’s functional plasticity, as revealed by BOLD fMRI technique, has the potential to elucidate the impact of different types of career training or work experience. However, there is still a lack of knowledge concerning how career training and experience is associated with the dynamics of resting-state functional connectivity, which we seek to address in this study.

### Dynamic Functional Connectomes and Brain States

Brain functional connectomes constructed using fMRI data depict the macroscale functional connectivity within the brain (Van Dijk et al., 2010; Sporns, 2011) and have been shown to be powerful in differentiating brain conditions (Lynall et al., 2010). A growing number of reports has also suggested that the brain’s functional connectome under resting or task conditions is not static but exhibits complex spatiotemporal dynamics as the brain undergoes dynamic integration, coordination, and responses to internal and external stimuli across multiple time scales (Chang and Glover, 2010; Hutchison et al., 2013; Calhoun et al., 2014). This phenomenon potentially implies that the time-varying dynamic functional connectome (DFC) could offer a more complete description of brain activity in comparison to the static one. For example, Leonardi et al. (2013) used principal component analysis to analyze the DFC in a healthy control group and a disabled relapse-remitting multiple sclerosis (RRMS) group and identified significant connections centered in the default mode network (DMN) with altered contributions in patients. Furthermore, taking advantage of the intrinsic connectivity networks and the corresponding time courses generated by the group ICA (Calhoun et al., 2001), Yu et al. (2015) first constructed DFCs using the intrinsic connectivity networks as nodes and the correlation of sliding time-windowed time courses as edges; then, dynamic graph metrics, such as connectivity strength, clustering coefficients, and global efficiency (Rubinov and Sporns, 2010), were calculated for the healthy group and the schizophrenia group, respectively. Their results demonstrated that the aforementioned measurements in the schizophrenia patients exhibited the lower variances over time in contrast to the healthy group, which provided a new perspective on the pathogenesis of schizophrenia. In addition, based on the brain’s dynamic transition and the Fisher discrimination dictionary learning (FDDL) technique (Yang et al., 2014). Zhang et al. (2013) proposed a novel dynamic brain functional connectome characterization model (DBFCC), which successfully extracted the representative atomic connectome patterns (ACP) for the resting and task-related conditions, respectively. Similarly, Li et al. (2014) applied DBFCC to characterize and differentiate the dynamic brain states in the healthy group and the post-traumatic stress disorder group, which also effectively generated two distinct ACPs for the post-traumatic stress disorder group. All of the above studies suggested that DFCs with time-varying information could provide a more accurate description of the brain’s activity.

### Study Purpose

Inspired by the aforementioned studies, we inferred that the spatiotemporal properties of the DFCs on a finer time scale could potentially provide us with evidence of the relationship between resting-state functional connectivity and career training or long-term experience. The DBFCC model (Zhang et al., 2013; Li et al., 2014) had the advantage of characterizing and differentiating the brain states. However, DBFCC appeared to be vulnerable to the random cluster center selection in K-Means for the clustering of WQCP (whole-brain quasi-stable connectome pattern) samples, which led to randomness and instability in the clustered results. In order to overcome the aforementioned deficiency in DBFCC, the automatic target generation process (ATGP) (Ren and Chang, 2003; Chang et al., 2011) based K-Means clustering for WQCP samples was proposed. Then, to further investigate the relationship between the brain dynamics of resting-state functional connectivity and extensive career training or experience, taking the sailors as an example, we applied the DBFCC with ATGP-K-Means clustering to explore the association between the DFC and sailing experience.

Generally, the seafarers are suitably used to explore the relationship between brain dynamics and career experience due to their occupational stability and professional particularity. For example, a sailor’s occupation requires a certain degree of particularity due to the following influencing factors: (1) the marine working environment, a small working space with machinery noise, single-sex colleagues (all male sailors), and long periods of isolation from their families; (2) requirements for good psychological health and strong environmental adaptability; (3) the required maritime professional skills; and (4) strong execution of behavior in a chain of command. Due to the particularity of a sailor’s occupation, we speculate that the long-term sailing experience and career training of seafarers will alter the temporal features of the functional connections among the relevant brain regions, which help to characterize the intrinsic neural substrates of career experience. Also, some common functional connections among specific brain regions regardless of sailing experience, are also maintained as the baseline of dynamic brain function. Thus, we will test this hypothesis as described in the following sections.

The remainder of this paper is organized as follows: the materials’ information and framework of DBFCC with ATGP-K-Means clustering are first presented; next, DBFCC is used to explore the common/distinct ACPs between the sailor and non-sailor groups. Finally, the results and analysis are presented together with interpretations, discussion, and conclusions related to the sailors’ career training and experience.

## Materials and Methods

### Data Acquisition and Data Preprocessing

Twenty male seafarers [ages: 42–57 years, mean age = 49 years; right handedness] with various positions, such as mate, helmsman, and seaman, were recruited from a shipping company in Shanghai, China. All sailors had approximately 10–20 years of sailing experience. For the non-sailor group, 20 male Chinese participants with matched ages in contrast to the sailors [ages: 48–55 years, mean age = 51 years; right handedness] who worked on land were recruited. All participants were informed about the purpose of this study and signed the written consent form according to procedures approved by the IRB of East China Normal University (ECNU), and none of the participants had a history of neurological and psychiatric disorders. Additionally, all participants did not exhibit abnormalities in the Symptom Checklist-90 (SCL-90) evaluation (Derogatis et al., 1976). The education levels for all the participants were junior college or equivalent, which were matched for two control groups. In data acquisition stage, all participants were instructed to wear ear plugs and to keep their body motionless with their eyes closed, remaining relaxed and awake. The corresponding resting-state BOLD fMRI data for each subject was scanned at the Shanghai Key Laboratory of Magnetic Resonance of ECNU. The concrete acquisition parameters were listed as follows: GE 3.0 Tesla, gradient echo EPI with 36 slices providing whole-brain coverage, number of time points = 160, TR (time of repetition) = 2 s, matrix size = 64 × 64, in-plane resolution = 3.75 mm × 3.75 mm, and slice thickness = 4 mm.

The preprocessing steps for the resting-state fMRI data included slice timing, head motion correction, nuisance covariate (six parameters related to head movement, white matter, and CSF signals) regression, spatial normalization, spatial smoothing with Gaussian kernel of 4 mm, and temporal filtering (0.01–0.08 Hz). After the preprocessing procedure, Craddock’s brain atlas with 200 ROIs (region of interest) (Craddock et al., 2012) was used to extract the time series of each ROI for each subject; then, the mean time series of each ROI was used as the reference for each ROI. Craddock’s brain atlas is a whole-brain functional atlas established by fMRI data, which seemed to be more suitable to accurately describe brain function than the widely used structural brain atlas, e.g., automated anatomical labeling template (Tzourio-Mazoyer et al., 2002).

### Framework of DBFCC

**FIGURE 1:**
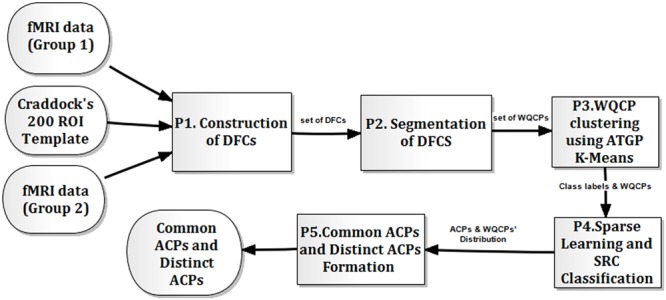
The flow chart of dynamic functional connectome characterization (DBFCC) model with ATGP-K-Means clustering.

The DBFCC with ATGP-K-Means clustering consisted of five sub-procedures (see P1–P5 depicted in **Figure 1**). The first procedure (P1) was the construction of DFCs, which was based on the Pearson correlation for the sliding time window constrained paired reference time series for any two ROIs in two control groups, i.e., Group 1 and Group 2, depicted in **Figure 1**. Then, the manual segmentation of DFCs (P2) was involved, forming the WQCP sample set (Zhang et al., 2013), which was described in detail in Section “Formation of WQCP Samples.” Thirdly, the ATGP-K-Means clustering was proposed to obtain the class labels for each sample in a WQCP set (P3), which eliminated the resulting randomness of the clustering of K-Means in the original DBFCC (Zhang et al., 2013; Li et al., 2014) (see Section “ATGP-K-Means Clustering on WQCP Set”). Furthermore, the FDDL method (Yang et al., 2014) and the sparse representation based classification (SRC) (Wright et al., 2009) were utilized to generate the ACPs and determine the distributions of the WQCP samples corresponding to each control group under each dictionary class (P4) (see Section “Sparse Learning and SRC Classification”). Finally, the common ACPs and distinct ACPs were classified, based on the ratio distributions of WQCP samples from two groups under the sub-dictionaries generated by FDDL (P5) (see Section “Formation of Common/Distinct ACPs”).

### Formation of WQCP Samples

The functional connectivity undergoes temporal dynamic transitions (Chang and Glover, 2010; Majeed et al., 2011; Smith et al., 2012; Hutchison et al., 2013; Calhoun et al., 2014), implying that a DFC with more abundant information is better to describe the brain’s functional topology than a static one does. Thus, the DFC analysis was adopted in this study. For convenience, we denoted the reference time series for the *i*th ROI of a specific resting-state fMRI data from Group 1 or Group 2 as $\bar{TS}$_i_, 1 ≤ i ≤ 200. Specifically, for a given time point *t*, the functional connectivity between the temporal segments *W_i_* and *W_j_* regarding $\bar{TS}$_i_ and $\bar{TS}$_j_ was defined as:

(1) $$\begin{array}{c} FC_{i,\;j,\;t}{= abs(correcoef(W}_{i}{,W}_{j)),}\;\\ FC_{i,\;j,\;t}=0\;\mathrm{if}\;i=j,i,j\in [1,\cdots ,200], \end{array}$$

where W_i_ = [$\bar{TS}$_i,t_,$\bar{TS}$_i,t+1_, ...,$\bar{TS}$_i,t+l-1_], W_j_ = [$\bar{TS}$_j,t_, $\bar{TS}$_j,t+1_, ..., $\bar{TS}$_j,t+l-1_], and *l* was the length of the sliding time window (*l* = 15 with an empirical setting). As the time window slid along the time axis, the DFC was formed as a three-dimensional matrix *DFC* with dimensions of 200 × 200 × (*L*-*l*+ 1), in which *L* denoted the length of $\bar{TS}$_i_.

**FIGURE 2:**
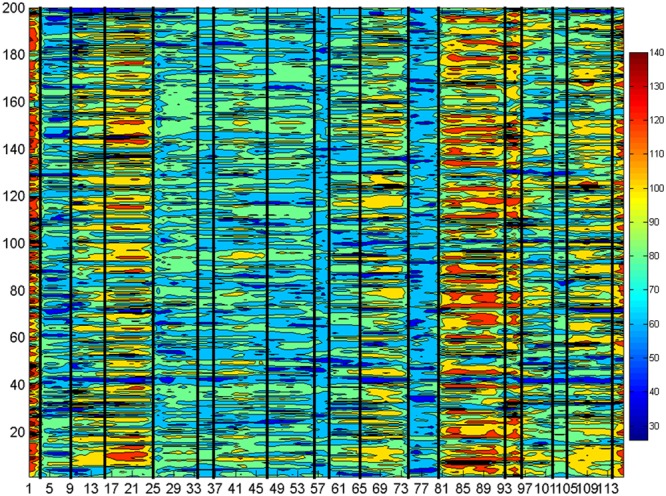
The FCS of the dynamic functional connectome (DFC) for a resting-state functional magnetic resonance imaging (fMRI) sample data.

According to prior studies (Zhang et al., 2013; Li et al., 2014), the functional cumulative connectome strength (FCS) was defined as follow:

(2) $$\begin{array}{c} FCS_{i,\;t}=\sum_{j\;=\;1}^{200}DFC_{i,\;j,\;t}, \end{array}$$

where *FCS* was a matrix with dimensions of 200 × (*L* -*l* + 1), and one sample of *FCS* was depicted in **Figure 2**, in which the horizontal axis represented the time points and the vertical axis denoted the cumulative connectivity strength of each ROI from Craddock’s atlas. As shown in **Figure 2**, we found that the FCS consistently maintained a stable connectivity strength in a short period along the horizontal axis. Considering this, we manually divided the whole FCS for each resting-state fMRI data along the time axis into quasi-stable segments (see the dividing black line in **Figure 2**) as suggested in Zhang et al. (2013) and Li et al. (2014), forming the set of WQCP samples.

### ATGP-K-Means Clustering on WQCP Set

Each WQCP sample in the WQCP set from Group 1 and Group 2 belonged to different classes, represented by ACPs, which revealed both similar and different functional topologies of the brain dynamics in two control conditions. In DBFCC, the original K-Means clustering was firstly used to categorize the WQCP samples into different classes and each WQCP sample’s class label was further retrieved. However, the performance of K-Means was greatly impacted by the randomness of the initial cluster point selection. Thus, in this study, the ATGP algorithm (Ren and Chang, 2003; Chang et al., 2011) was applied to determine the initial cluster points, forming the ATGP-K-Means clustering algorithm. The effectiveness of ATGP in initialization was validated in our previous studies, overcoming the randomness of FastICA (Hyvärinen, 1999; Yao et al., 2013) and improving the accuracy of the separation of brain sources in SDLS (Wang et al., 2016a).

### Sparse Learning and SRC Classification

The sparse representation has applied for signal processing, noise suppression (Elad and Aharon, 2006), pattern classification (Huang and Aviyente, 2006), and signal reconstruction (Bruckstein et al., 2009). However, for classification using sparse representation, Huang and Aviyente (2006) noted that reconstructing the signal accurately was not sufficient, while the discrimination of the given signal classes was also important. Based on this idea, the FDDL methodology (Yang et al., 2014) was adopted in this paper to determine the ACPs of resting brain states from the WQCP set. Generally, FDDL fully considered the accuracy of signal reconstruction, the discrimination among the sub-dictionaries and the discrimination of sparse coding coefficients. According to Zhang et al. (2013), FDDL performed better in the determination of ACPs in the WQCP samples than the online sparse dictionary learning algorithm (Mairal et al., 2010). Assuming that all the WQCP samples in two control groups were arranged in a matrix, denoted *X*, and that ATGP-K-Means clustering had separated the WQCP samples in *X* into *c* classes, the FDDL was expressed mathematically as follow:

(3) $$\begin{array}{c} J_{\left(D,\;S\right)}=\arg\underset{\left(D,\;S\right)}{\min}\{r(X,D,S)+\lambda_{1}||S|{|}_{1}+\lambda_{2}f(s)\}, \end{array}$$

where the first term *r*(*X, D, S*) was the constraint on discriminative fidelity, the second term indicated sparsity constraint, and the last term represented Fisher discriminative constraint of the sparse coding coefficients. Furthermore, *r*(*X, D, S*) was expressed as:

(4) $$\begin{array}{c} r(X_{i},D,S_{i})=\;||X_{i}-DS_{i}||{}_{F}^{2}+||X_{i}-D_{i}S_{i}^{i}|{|}_{F}^{2}+\sum_{\begin{array}{c} j\;=\;1\\ j\;\neq \;i \end{array}}^{c}||D_{j}S_{i}^{j}|{|}_{F}^{2}, \end{array}$$

where S_i_ = [S_i_^1^; ... ; S_i_^j^; ... ; S_i_^c^], S_i_^j^ denoted the coding coefficient of *X_i_* over *D_j_*. In equation (4), the first term was used to enforce the dictionary *D* with good representative ability for *X_i_*; meanwhile, the other two terms were utilized to showed that the sub-dictionary *D_i_* expressed *X_i_* and the other sub-dictionaries were less to express *X_i_*. Further, the constraint term *f*(*S*) was formulated as follows:

(5) $$\begin{array}{c} f(S)=tr(S_{W}(S))-tr(S_{B}(S))+\eta ||S|{|}_{F}^{2}, \end{array}$$

(6) $$\begin{array}{c} S_{W}(S)=\sum_{i\;=\;1}^{c}\sum_{s_{k}\;\in \;S_{i}}(s_{k}-m_{i})\;{\left(s_{k}-m_{i}\right)}^{T}, \end{array}$$

(7) $$\begin{array}{c} S_{B}(S)=\sum_{i\;=\;1}^{c}n_{i}(m_{i}-m)\;{\left(m_{i}-m\right)}^{T}, \end{array}$$

where *m_i_* and *m* represented the mean vectors of *S_i_* and *S*, respectively, and *n_i_* indicated the sample number of class *X_i_*. The discriminative coefficient term *f*(*S*) here made the dictionary discriminative for the training samples, and was achieved by minimizing the within-class scatter *S_W_*(*S*) and maximizing the between-class scatter *S_B_*(*S*) of *S*. The parameter η was set to 1 for both algorithms’ convexity and maximizing the discriminability as described in Yang et al. (2014). The values of the parameters λ_1_ and λ_2_ were set to 0.01 and 0.02 according to Li et al. (2014), respectively.

In DBFCC, the SRC algorithm (Wright et al., 2009) was used to classify the testing WQCP sample, e.g., *x_test_*, which was expressed as

(8) $$\begin{array}{c} {\hat{s}}_{test}=\arg\underset{s}{\min}\left\{||x_{test}-Ds|{|}_{2}^{2}+\lambda ||s|{|}_{1}\right\}, \end{array}$$

(9) $$\begin{array}{c} ind=\arg\underset{i}{\min}\{e_{i}\}, \end{array}$$

where e_i_ = ∥x_test_ - D_i_ŝ_i_ ∥_2_, ŝ = [ŝ_1_;...;ŝ_i_;...,ŝ_c_] and ŝ_i_ represented the coefficient vectors under the *i*th sub-dictionary class. Further, in both ATGP-K-Means and FDDL, the Bayesian information criterion (BIC) (Schwarz, 1978) was used to estimate the number of clusters (*c*), which was also formulated in previous studies (for details, see Zhang et al., 2013; Li et al., 2014).

### Formation of Common/Distinct ACPs

Using sparse learning with FDDL for the WQCP sample matrix *X*, the corresponding dictionary *D* was extracted, including *c* sub-dictionaries. Then, the SRC algorithm was used to classify the WQCP samples for each control group, i.e., *X_G_*_1_ or *X_G_*_2_, which classified each WQCP sample into a certain sub-dictionary *D_i_* (1 ≤*i* ≤*c*). Furthermore, all of the DFCs corresponding to each WQCP for each sub-dictionary (*D_i_*,1 ≤*i* ≤*c*) were retrieved, and the corresponding mean functional connectome along the time dimension was calculated, which formed the ACPs with a dimension of 200 × 200 for each sub-dictionary *D_i_* (1 ≤ *i* ≤*c*). Finally, the *c* ACPs from Group 1 and Group 2 were generated, respectively.

All the above formed *c* ACPs could be divided into common ACPs (representing the common brain states) and distinct ACPs (denoting the distinct brain states) regarding two control groups, based on the ratio distributions of WQCP samples from two control groups under the sub-dictionaries generated by FDDL (Li et al., 2014). Namely, some WQCP samples which formed the distinct ACPs, only mostly existed in one certain group. Furthermore, the ACPs should yield high reproducibility and reliability among the resampled WQCP sample sets, i.e., the resampled matrix *X_res_*, which could validate effectiveness of the division of common/distinct ACPs. The idea of validation process was simple but heuristic: first, the multiple WQCP subsets (*X_res_*) with a half scale in contrast to the WQCP set (*X*) were generated by the randomly repeated resampling of the WQCP set; then, the FDDL and SRC algorithms were used to perform the sparse learning and classification tasks, respectively, which generated the ratio distributions of the WQCP samples under the variant sub-dictionaries and the corresponding ACPs for each control group; finally, the ratio distributions of the WQCP samples under sampling process for each group could be used to check the previous division of common and distinct ACPs.

## Results and Analysis

### The Ratio Distributions of WQCP Set under Resampling Process

**FIGURE 3:**
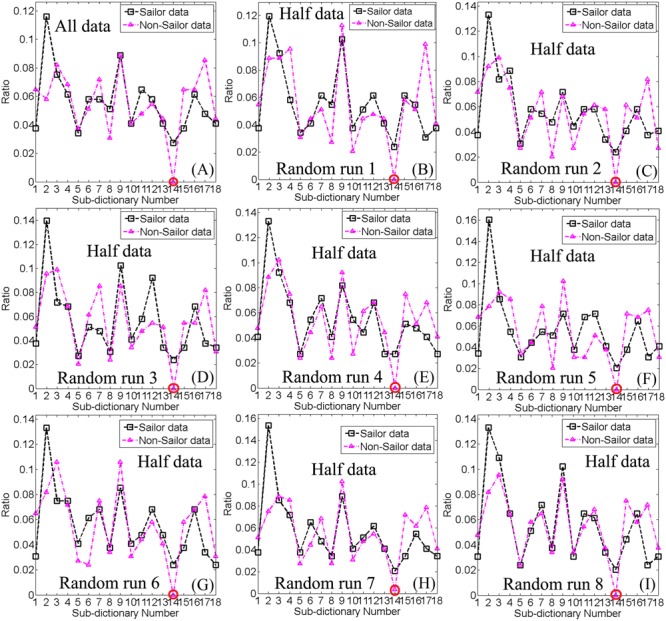
**(A)** The ratio distributions of WQCPs for the sailor and non-sailor groups under non-resample process; **(B–I)** The ratio distributions of WQCPs for the sailor and non-sailor groups under eight instances of resampling process, respectively. The horizontal axis represents the index of each sub-dictionary, and the vertical axis denotes the ratio distribution of WQCPs under different sub-dictionaries. The circled number on the *x*-axis represents the indexes of distinct atomic connectome patterns (ACPs) between the sailor and non-sailor groups, while the rest denote the indexes of the common ACPs of the two groups.

**Figure 3** depicted the ratio distributions of the WQCP samples corresponding to each control group (sailor group or non-sailor group) under 18 sub-dictionaries generated by FDDL. Specifically, **Figure 3A** showed the ratio distributions of the WQCP samples corresponding to the sailor group or non-sailor group using all WQCP samples for FDDL dictionary learning, while the rest of the sub-figures (**Figures 3B–I**) demonstrated the corresponding sailor group’s or non-sailor group’s ratio distributions of WQCP samples under eight randomly resampling process. As shown in **Figure 3**, we found that the 18 sub-dictionaries determined by FDDL in both the subsampled WQCP set (*X_res_*) and the whole WQCP set (*X*) exhibited very strong reproducibility and reliability. By observing the ratio distributions of the WQCP samples of each control group in **Figure 3**, we further found that there existed 17 common ACPs and one distinct ACP. Among the 17 common ACPs, the ratio distributions of ACP12 had most consistent difference, implying that compared with the non-sailors, the sailors had a better chance of staying a brain state related to ACP12 as the brain activity went on. This phenomenon demonstrated that the career training with a long period could possibly shape the chances of the brain states where the brain activity belonged to. Besides, the index of the distinct ACP was 14, circled in red along the *x*-axis in each sub-figure of **Figure 3**, as indicated by the significant difference in the ratio distributions for sailor group and non-sailor group. It was noteworthy that the distinct ACP14 exhibited a unique pattern only in the sailor group, and had the potential to play a specific role in this group.

### Common/Distinct ACPs

**FIGURE 4:**
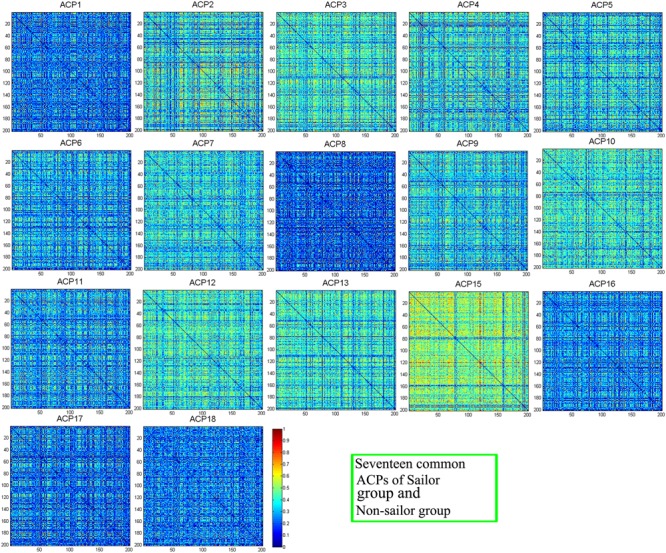
Seventeen common ACPs with a full matrix view for the sailor and non-sailor groups.

**FIGURE 5:**
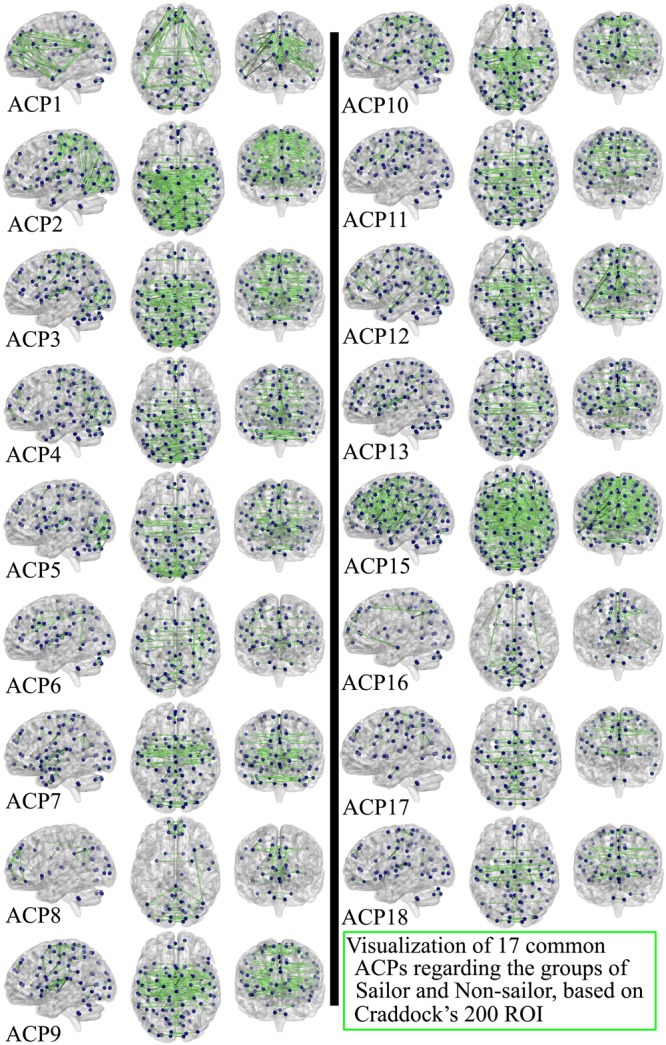
Seventeen common ACPs for the sailor and non-sailor groups, projected onto a standard brain surface. Only the connective edges with strength greater than 0.75 were retained.

**Figure 4** showed the seventeen common ACPs with a full matrix view for both sailor and non-sailor groups. Correspondingly, the 17 common ACPs were projected onto a standard brain surface, as shown in **Figure 5**. Specifically, the connective edges in all common ACPs were retained with a strength larger than 0.75, where the threshold value was also applied in Li et al. (2014).

**FIGURE 6:**
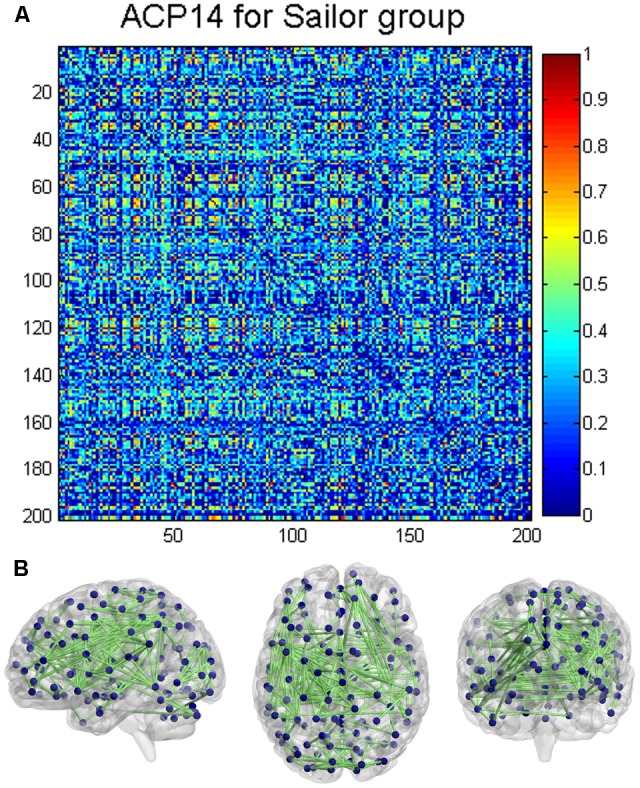
Distinct ACP (ACP14) only existed in the sailor group. **(A)** ACP14 with a full matrix view; **(B)** ACP14 projected onto a standard brain surface. Only the connective edges with strength greater than 0.75 were retained.

**Figure 6A** depicted the distinct ACP14 with a full matrix view, only for the sailor group, while **Figure 6B** showed the connectivity patterns of the distinct ACP14 with strength greater than 0.75, projected onto a standard brain surface. It was noteworthy that ACP14 only existed in the sailor group, which was possibly closely related to the career training and long-term offshore operation of the sailors in contrast to the non-sailors, which was discussed in Section “Distinct ACPs.”

## Discussion

### Common ACPs

In this study, 17 networks were classified as the common ACPs among the sailors and non-sailors (shown in **Figures 4, 5**), which represented the common functional topology for both control groups. A very important functional network, called DMN, was found in the resting human brain, in which the abnormal or disconnections were often observed with the neuropsychiatric disorders. The DMN was regarded as the potential biomarkers of neuroimaging in many brain diseases, such as Alzheimer’s disease (Greicius et al., 2004), mild cognitive environment (Petrella et al., 2011), depression (Sheline et al., 2009), and schizophrenia (Garrity et al., 2007). According to Raichle et al. (2001), the involved regions include medial prefrontal cortex (MPFC), posterior cingulate cortex (PCC), precuneus, and parietal cortex. The superior frontal cortex, parahippocampal gyrus, inferior temporal cortex, retrosplenial cortex, and cerebellar tonsils could also be considered to be the seed regions for DMN (Fair et al., 2008). On the whole, the common ACPs covered the regions of DMN but there were differences among them, which implied that the DMN functions as a network hub (Liang et al., 2013) in dynamic brain transitions. ACP15 exhibited the highest degree of complexity and the largest number of connections of all brain regions, which justifies why the brain represents 2% of the body’s weight but consumes approximately 20% of the calories for the entire body (Raichle and Gusnard, 2002; Zhang and Raichle, 2010). We inferred that ACP15 may represent part of the brain’s ongoing activity, which not only supports the maintenance of the neurons’ responsiveness for the transient and ever-changing functions of the brain but also initiates sustained functionality (Raichle and Gusnard, 2002). ACP1 was the most similar to DMN with direct connections among the PCC, temporal cortex, precuneus, and MPFC, but was more sparse than ACP12, which was also involved in many connections in the occipital cortex. This provides possible evidence of the exchange of information between DMN and visual network during scanning. Additionally, ACP1 also showed that the DMN exhibited high reproducibility in dynamic brain transitions. In ACP2, ACP3, ACP4, and ACP5, the connections were mostly present in the areas of the parietal lobe and occipital lobe, but ACP4 exhibited connections to the inferior temporal cortex and MPFC. The connections of the two hemispheres were not symmetrical, similar to ACP6, with more connections on the right side. More connections were present on the left side in ACP16 and ACP10. Furthermore, ACP6, ACP8, and ACP16 exhibited more connections within these regions, such as MPFC, precuneus, occipital, and temporal lobes, but were more sparse compared to the rest ACPs. In addition, the connections of ACP7, ACP9, ACP17, and ACP18 mainly covered the sensorimotor, visual, and temporal networks. Finally, regions in the cerebellum were mostly connected with regions in the occipital lobe such as ACP3, ACP5, ACP6, ACP15, and ACP16, while the others exhibited connections inside cerebellum itself.

### Distinct ACPs

Using DBFCC model, we identified one distinct ACP, i.e., ACP14 (depicted in **Figure 6**), which obviously denoted different functional topology for both control groups. ACP14 was a characterized ACP of the sailors in contrast to the non-sailors. Many sub-networks were involved in ACP14, such as the auditory network, visual network, executive control network (Beckmann et al., 2005; Damoiseaux et al., 2006; Wang et al., 2012), and vestibular function-related network (Highstein et al., 2004; Angelaki and Cullen, 2008), which implied its complexity and the potential relationship to the seafarers’ career experience and professional particularity as discussed below. The auditory network may be associated with the continuous noise disturbances of machines running and ocean sounds. The visual network covered both the basic and advanced visual regions, which possibly implied that the complex sea conditions enforced the combination of the fundamental visual cortex’s discovery function and the senior visual cortex’s judgment function. The specific functional connections of the basic visual regions and the advanced regions in contrast to the non-sailor group may imply the high efficiency of the dynamic information adjustment of visual circuits. The vestibular function-related network was closely related to the seafarers’ occupational training and long-term offshore operations, in which the co-regulatory role of the vestibular system and the visual network allowed the sailors to maintain their body balance and clear vision and improve the ability to determine their own position in three-dimensional space in the maritime environment (Highstein et al., 2004; Angelaki and Cullen, 2008). The executive control network was possibly enhanced by the semi-military training and management (e.g., obeying orders from the captain) of the sailors in the career training stage and the offshore operation stage, which consistently modulated the brain activity of the executive related cortex regions. In summary, the occupational training and long-term offshore experience of the sailors could reorganize the topology of the brain’s functional networks in order to accomplish the long-term operation at sea in contrast to the non-sailors on land, which demonstrated the flexibility in the human brain’s functional plasticity.

### Limitations and Future Work

There are certain limitations to this study. First, we only considered sailors with long-term sailing experience in excess of 10 years, limiting the number of subjects. Thus, future studies with a larger number of sailors would more definitively explore the functional plasticity driven by career sailing experience. Further, the characterized ACP14 was identified in the sailors with long-term sailing experience. However, whether this factor depended on the duration of sailing experience should be further investigated.

## Conclusion

The DBFCC model with ATGP-K-Means clustering was effective to characterize the common and different topology of the DFC in sailors and non-sailors. Firstly, the reproducible common ACPs shared by the sailors and non-sailors implied that common dynamic transition states existed, possibly as the functional transition baseline of the human brain. Furthermore, we found that the brain’s functional plasticity could be modulated by the longer-term career sailing experience. Stated concretely, one reproducible distinct ACP of the sailors in contrast to the non-sailors showed a close relationship to long-term sailing training and experience, which was the potential to be as a biomarker to characterize the sailors’ functional brain. Our findings provide the evidence of that how the sailing experience could influence the dynamic functional reorganization in the healthy human brain to satisfy the professional particularity. Also, this study demonstrates the effectiveness of the revised DBFCC model, which potentially has wide applicability in the exploration of the functional plasticity driven by other types of career training and experience.

## Author Contributions

Collection of fMRI data: NW, WZ, and YS. Design of the work: NW, HY, and WZ. Analysis and interpretation: NW, HY, WZ, and YS. Drafting the article: NW, HY, and WZ.

## Conflict of Interest Statement

The authors declare that the research was conducted in the absence of any commercial or financial relationships that could be construed as a potential conflict of interest.
